# Efficacy of teduglutide in a patient with Crohn’s disease and short bowel syndrome on enteral nutrition: let’s start to think out of the box

**DOI:** 10.1093/gastro/goz030

**Published:** 2019-07-13

**Authors:** Brigida Barberio, Giacomo Carlo Sturniolo, Renata D’Incà, Fabio Farinati, Maria Assunta Bigotto, Matteo Ghisa, Greta Lorenzon, Edoardo Savarino

**Affiliations:** 1 Department of Surgery, Oncology and Gastroenterology, University of Padua, Padua, Italy; 2 Department of Pharmaceutical Sciences, University of Padua, Padua, Italy

## Introduction

Short bowel syndrome (SBS) is a disorder characterized by malabsorption due to the absence or resection of a long small intestine tract, with an incidence of 2 per million of habitants. In adults, SBS is defined as less than 200 cm without colon and less than 50 cm with residual colon in continuity [1]. The causes of SBS in adult patients are different, but the most frequent one (33.3% of the cases) is the need for surgery in patients with Crohn’s disease (CD) [2].

Teduglutide (TED) is a drug analogous to glucagon-like peptide 2 (GLP-2), recently approved for the treatment of adult patients with SBS on parenteral nutrition, with a dosage of 0.05 mg/kg subcutaneous injection once daily [3]. Currently, medical literature does not report clinical studies or case reports evaluating the use of TED in patients affected by SBS on enteral nutrition due to the off-label indication.

## Case presentation

We report a case of a 65-year-old woman affected by a severe form of CD, diagnosed in 1991. From the beginning, the disease had an ileocolonic localization with a stricturing-fistulizing phenotype. Because of the extensive and aggressive disease, together with the lack of response to conventional and biological treatments (i.e. Infliximab, Adalimumab, salazopyrine, multiple cycles of systemic steroids and antibiotics), the patient underwent multiple abdominal surgical resections, the last one in 2010, with a final ileo-colonic anastomosis. Overall, the remaining gut was of about 110 cm in length. Thus, the patient developed a SBS, although she did not required parenteral nutrition.

In November 2015, while she was under treatment with Adalimumab, she reported from 20 to 40 bowel movements per day with watery stools and progressive loss weight with a body mass index of 17 kg/m^2^. Due to significant electrolyte losses and recurrent metabolic acidosis, the patient underwent biweekly infusions of magnesium, potassium, and bicarbonate in addition to oral supplementation. Moreover, she was supplied with enteral nutrition, since oral nutrition resulted in worsening of the diarrhea.

In March 2016, after an ineffective attempt with octreotide, the patient was treated with TED 0.05 mg/kg daily, although the drug was approved only for patients with SBS on parenteral nutrition. However, after 24 weeks of treatment with TED, enteral nutrition was reduced by at least 50% and, after 72 weeks, it was suspended permanently. Moreover, intravenous infusions of magnesium and potassium were nearly abolished (i.e. once every 3 months). The patient, currently under treatment, is on oral feeding and is reporting four evacuations/day with formed stools. During treatment, the patient did not develop mild side effects, except for a diffuse abdominal pain and nausea, occurring only during the first week of treatment. No serious side effects were recorded during the whole period. Of note, the therapy markedly improved the quality of life of the patient, resulting in a normal social and private life.

## Discussion

Symptoms and severity of SBS depend on the anatomic sections of the intestine that are resected, the length and absorptive capacity of the remnant bowel, and the presence of active primary disease [4]. Patients who underwent ileal resections have a higher possibility of experiencing diarrhea compared to patients with jejunal resections because the distal small intestine has a lower capacity to reabsorb water compared to the colon. Diarrhea is exacerbated in case the colon is partially resected, as well [5].

Our patient had an ileocolic anastomosis (left colon in continuity), without ileocecal valve, with multiple intestinal resections and a remaining small bowel of about 110 cm. These are the patients who exhibit the greatest risk for development of dehydration and malnutrition. Ileal resection also reduces the plasma concentration of various digestive hormonal mediators such as GLP-1, GLP-2, and Peptide YY, all synthesized by enteroendocrine L cells in the distal ileum and colon [6].

TED, acting similarly to GLP-2, increases small intestinal mucosal growth by enhancing crypt cell growth and inhibiting apoptosis; also, it decreases gastric motility and reduces gastric secretion, increases mesenteric blood flow, and supports the gut barrier function in animal and human studies [3]. The open-label, non-placebo-controlled pilot study by Jeppesen *et al.* [7] first evaluated the safety and efficacy of TED in 16 SBS patients. When compared to the baseline values, TED increased wet weight absorption, urine weight, and urine sodium excretion. Moreover, TED decreased faecal wet weight and faecal energy excretion. In SBS patients with end jejunostomy, TED increased villus height (*P *=* *0.030) significantly, crypt depth (*P *=* *0.010), and mitotic index (*P *=* *0.010). Afterwards, a first long-term (24-week) randomized placebo-controlled study (STEPS-1) of TED in 83 patients with SBS on parenteral support was published. Responders were patients who reduced by >20% in parenteral volumes from baseline at Weeks 20 and 24 (63% of patients) [8]. The long-term STEPS-2 study, the 2-year open-label extension study, and STEPS-3, the 1-year open-label study, showed the effectiveness and the long-term safety of the treatment [9, 10]. Data from early reports on the short-term TED study suggested that the beneficial effects of TED are reversible within 3 weeks after the end of the treatment. This suggests that chronic long-term treatment is probably necessary to maintain the response. In keeping, we have to report that having our patient interrupt the treatment for a month due to some difficulties in obtaining that, she reported a progressive increase in bowel movements similar to the period before the starting of TED therapy.

At the moment the literature does not report clinical studies or case reports evaluating the use of TED in patients affected by SBS on enteral nutrition due to the off-label indication. So far, this is the first case which demonstrated that this kind of treatment may allow a drastic reduction in daily evacuations, interruption of enteral nutrition, and improvement of quality of life to be obtained. Still under treatment, the patient did not developed serious side effects, confirming the long-term safety of the medication. Thus, TED could be a promising and effective therapy even in patients with SBS on enteral nutrition, and this case supports thinking ‘out of the box’ when clinical indication is present. By the way, further studies are mandatory to recommend the use of TED in patients not in parenteral nutrition as suggested by current guidelines.

## Authors’ contributions

Performance of the research and writing of the manuscript: B.B. and E.S. Clinical management of the patient: All authors. All authors read and approved the final manuscript.

## Conflicts of interest

The authors declare that they have no Conflict of interest.
